# A Kidney Transplant Support System for Patient-Clinician Shared Decision-Making

**DOI:** 10.1007/s10916-025-02175-2

**Published:** 2025-05-10

**Authors:** Yunwei Zhang, Marni Torkel, Samuel Muller, Germaine Wong, Jean Yang

**Affiliations:** 1 Sydney Precision Data Science, The University of Sydney, Sydney, NSW Australia; 2 School of Mathematics and Statistics, The University of Sydney, Sydney, NSW Australia; 3 Charles Perkins Centre, The University of Sydney, Sydney, NSW Australia; 4 School of Mathematical and Physical Sciences, Macquarie University, Sydney, NSW Australia; 5 Sydney School of Public Health, The University of Sydney, Sydney, NSW Australia; 6 Centre for Kidney Research, Kids Research Institute, the Children’S Hospital at Westmead, Sydney, NSW Australia; 7 Centre for Transplant and Renal Research, Westmead Hospital, Sydney, NSW Australia; 8 School of Mathematics, Statistics, Chemistry and Physics, Murdoch University, Murdoch, WA Australia

**Keywords:** Kidney, Transplant, Shared Decision Making, Kidney Transplant Support System, Wait Time

## Abstract

An optimal deceased donor allocation program requires a fair, ethical, and transparent algorithm to ensure efficient and effective allocation of deceased donor kidneys to recipients that will benefit most, by maximizing utility of the donor organs, but at the same time, ensuring all potential candidates have equitable access and equal opportunity to this scarce resource. In response to the increasing demand and the limited availability of donor organs, there has been a global concerted effort to increase the use of less optimal donor kidneys in suitable recipients. During this complex allocation process, organ acceptance decision-making is the final step. Transplant nephrologists are typically the gatekeeper of this process and make the ultimate decision regarding organ suitability for the intended patients. However, with considerable evidence suggesting the value of shared decision making, engaging patients, caregivers and their primary nephrologists prior to accepting the allocated organ, ensures the healthcare decisions align with the patients’ values and their preferences. To tackle this challenge, we developed a visualisation guided simulation-based tool to assist shared decision-making. We have shown that the three-dimensional clinical information required for organ acceptance can be represented using an intuitive and user-friendly interface. By utilizing our published allocation simulation process, simKAP, this decision support system called Kidney Transplant Support System has the capacity to forecast a sequence of potential kidneys offered to a candidate on the waiting list, with the provision of estimated waiting time, donor quality and the expected post-transplant patient survivals for each consecutive offer. Implementation of this tool may inform shared decision-making and reduce organ discards.

**Supplementary Information:**

The online version contains supplementary material available at 10.1007/s10916-025-02175-2.

## Introduction

An optimal deceased donor allocation system requires a fair, ethical, and transparent algorithm to ensure efficient and effective allocation of deceased donor kidneys to recipients that will benefit most by maximizing utility of the donor organs, and ensuring all potential candidates have equitable access and equal opportunity to this scarce resource. In response to the increasing demand and limited availability of donor organs, there has been a global concerted effort to increase the use of less optimal donor kidneys in suitable recipients. Patient preferences for deceased donor kidney allocation prioritize equity-focused principles including waiting time, medical urgency, and quality of life [14, 15]. However, these priorities can sometimes contrast with the utility-focused approaches, which emphasize maximizing overall transplant benefit. This highlights the critical need for an allocation algorithm to balance equity and efficiency, such as a fair and optimal distribution of donor kidneys. A risk-based allocation algorithm has been proposed and implemented in several countries such as in the United States (US), in the United Kingdom (UK) and more recently in Australia [3, 4], aiming to maximize the gain in number of life years and quality-adjusted life years through appropriately matching potential transplant candidates with allografts that have similar anticipated survivals [10]. The deceased donor kidney allocation process in Australia operates as a two-tier point-based system, where kidneys are initially allocated nationally before being distributed within each state. All candidates on the deceased waiting list are matched to every available deceased donor offered nationwide. An overall allocation score is calculated based on various characteristics, including age, waiting time, HLA matching with the potential donor, the candidate's residing state, and their sensitization status. The candidate with the highest score (the best match) will then be offered the donor's kidney. If the candidate and their transplant clinician decline this offer, the next highest-ranked candidate will receive the offer.

Previous research [5, 16] has indicated that compared to being on dialysis, transplantation using suboptimal donors, if suitably allocated, can improve survival and overall quality of life, albeit the magnitude of benefits is smaller than transplantation using quality donors. However, challenges arose from this approach. First, it is difficult to accurately predict post-transplant events including complications and graft loss in clinical settings using pre-transplant donor and recipient information as many of these factors may interact and influence outcomes. Second, clinicians are concerned [16] that transplantation with marginal donors may lead to premature graft loss and post-transplant complications such as delayed graft function, and therefore, the desired incremental survival and quality of life benefits compared to being on dialysis could not be attained. Consequently, clinicians and patients may refuse to accept these suboptimal donor kidneys but prefer to wait for a future better-quality donor kidney, thus this increases the non-utilization rates of recovered organs that may be otherwise suitable for the appropriate patients. Donor non-utilization rate, particularly for non-ideal kidneys in the United States is around 25% [9, 12], and approximately 10% in Australia for higher kidney donor profile index (KDPI) donor kidneys [17],ANZDATA report-2025. Donor kidney discard may lead to many consequences, such as increasing the number of patients on the deceased donor waiting list, reducing the transplantation rates and, consequently, higher rates of premature death on the waiting list (https://pubmed.ncbi.nlm.nih.gov/39288350/.) To guide evidence-based shared clinical-decision making, we have previously developed simKAP [18], a simulated evaluation framework under the current allocation algorithm as well as various hypothetical allocation algorithms. simKAP has been cross-validated internationally and externally, that considers not only the donor and recipients’ characteristics, but also includes a shared-decision making process in the modelling. Using this framework, we aimed to develop the clinical decision support system coined Kidney Transplant Support System (KTSS). This system guides clinicians and their patients to make informed decisions about deceased donor kidney acceptance. This individualized, web-based clinical decision-making tool will be presented as an interactive platform.

## Materials and Methods

We provide detailed material and methods in our supplementary file.

## Results

**Fig. 1:**
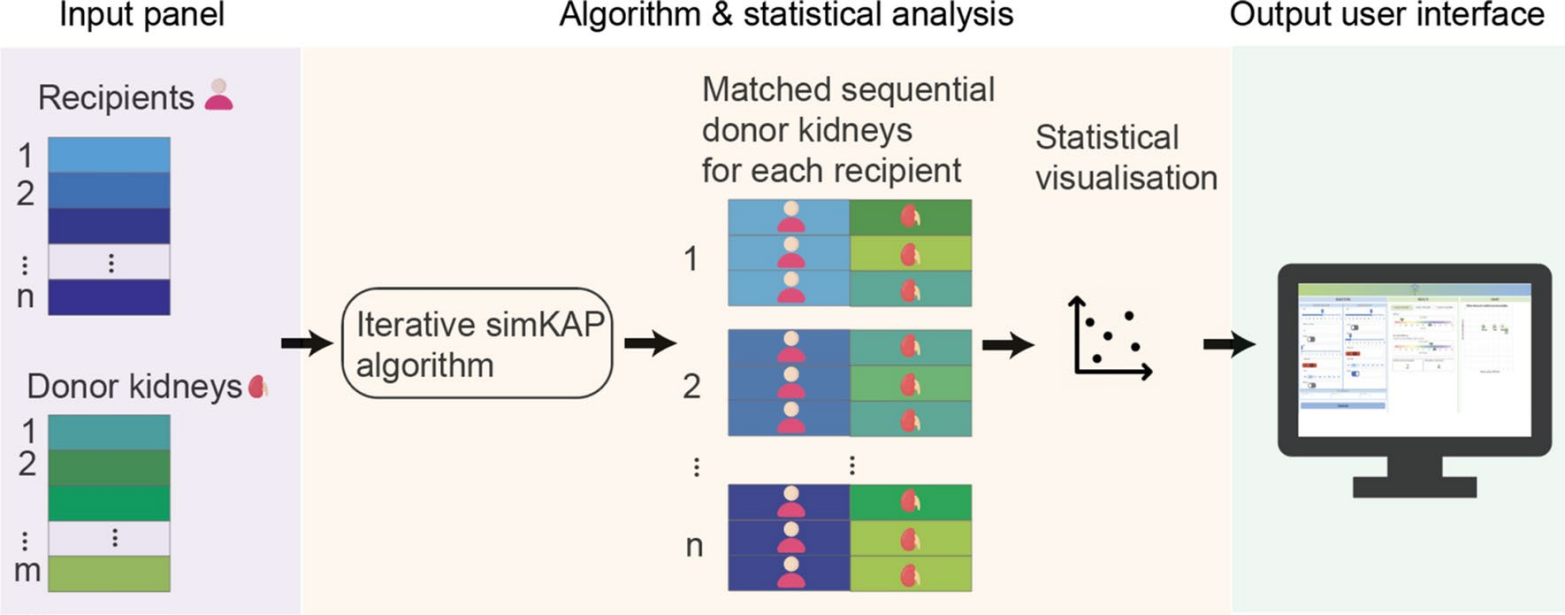
Workflow of the KTSS system. The KTSS system first requires recipients and donor kidneys’ characteristics as inputs in the input panel. The sequence of offered kidneys based on the simKAP algorithm is obtained, followed by survival model building and visualisation. Finally, we build a user interface to assist clinical decision-making

The Kidney Transplant Support System (KTSS) is an interactive web application designed to streamline the deceased kidney allocation process for transplant health professionals and patients alike (Fig. 1). The application combines user-centric design, accessibility, responsiveness and performance optimization to deliver a seamless and efficient user experience for patients and clinicians engaged in shared decision-making*.* Our tool dynamically adjusts to changing input data and is optimized for accessibility across various devices, including mobile phones, iPads, laptops, and desktop computers. The interactive platform comprises three primary panels: the selection, the results, and the chart panel, each serving a distinct function in facilitating informed decision-making.

**Fig. 2:**
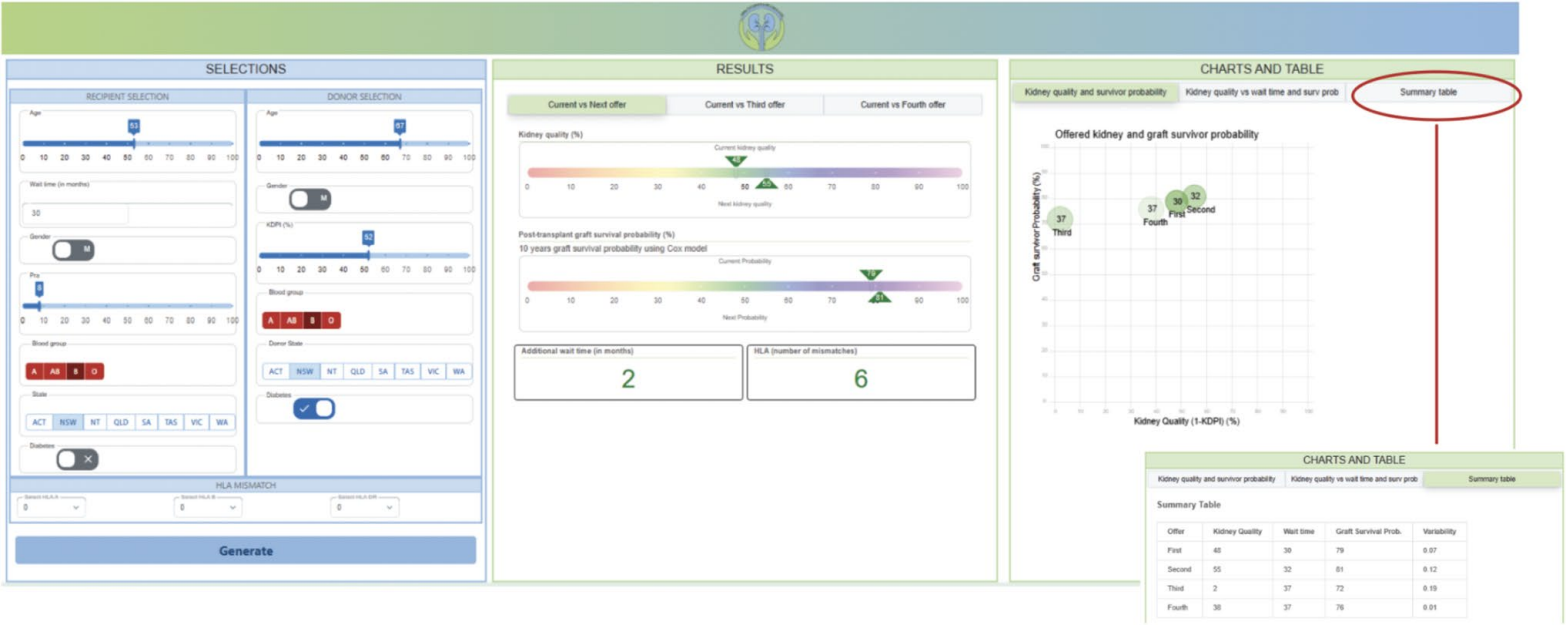
User interface of the Kidney Transplant Support System. The selection panel presents the user inputs, including the recipient and donor kidney characteristics. The results panel presents the comparison of the current offer up until the fourth offer, where the color bars show the kidney quality and predicted survival probability comparisons respectively and the bottom shows the wait time the recipient has to wait (from joining the waiting list until the * offer) and the HLA mismatching number. The chart panel presents a statistical graph an overview of all four offers. x-axis represents graft survival probability, y-axis represents kidney quality and the number in each dot represents the waiting time

**The “Selection” panel** positioned to the left, empowers clinicians to input recipient characteristics including recipient age at the time of offer, wait time, sex, blood group, presence of diabetes mellitus, panel reactive antibodies (PRA) level (an indication of the sensitization status of the recipient), and the recipient's current state; donor characteristics, including donor age, sex, kidney donor profile index (KDPI), blood group, donor’s state, presence of diabetes mellitus; and immunological measurements consisting of human leukocyte antigen (HLA) A, B and DR mismatches. As an example, let us consider a candidate recipient which is a 53-year-old male from NSW, Australia, with blood group B, PRA of 8, and no diabetes who have been offer a donor graft from a deceased 67-year-old diabetic male from NSW, Australia, with blood group B and donor KDPI of 52 percentiles. Figure 2a presents a screenshot of the selection panel with the recipient-based line info entered.

**The “**Results**” panel** positioned in the middle displays the outcomes generated by taking the input from selection panel and applying KTmatch to identify the closest match to this candidate. Here, the KDPI horizontal bar chart positioned on top, visually representing the KDPI values as percentages, with the top triangle indicating the current KDPI and the bottom triangle representing subsequent KDPIs. The second survival horizontal bar chart, positioned below the KDPI bar chart shows the predicted 10-year allograft survivals using the Cox model, with the small triangle at the top and bottom triangle indicating the current probability and the subsequent probabilities respectively. Positioned in the lower-left corner is the supplemental wait time incurred if the patient declines the current offer. Positioned in the lower-right corner is the total HLA mismatch for subsequent offers.

In the example above, the iterative simKAP algorithm simulated a hypothetical first four offers based on the transplant data from Australian and New Zealand from the last 11 years 2006–2017. The tab with “Current vs Next offer” under Results in Fig. 2 as an example shows that compared to the current offer, the next offer is with a better kidney quality (55%), a higher predicted 10-year graft survival probability of 81% and this candidate is forecasted to wait for an extra 2 months.

**Fig. 3:**
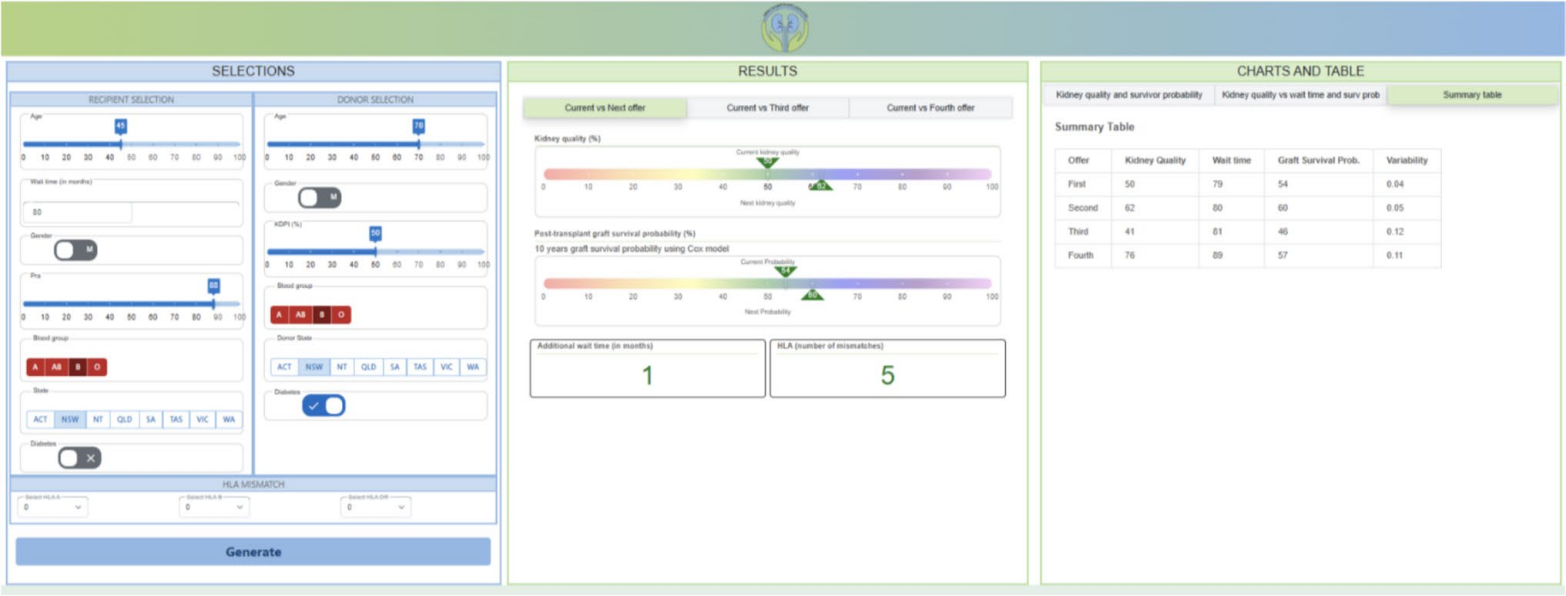
An example with highly sensitized candidate. The CHARTS AND TABLE panel presents the summarized table results for all four offers for this candidate

**The “Charts and Table” panel,** positioned to the right, presents a scatter plot, providing an overview of the first four simulated offers, consolidating crucial information that is essential for informed decision-making, as depicted on the far right of the Fig. 2. Here, we look at another example of a highly sensitized candidate (aged 45 years, with PRA more than 88%), and the current offer of a 70-year-old deceased donor with KDPI of 50% from interstate (Fig. 3). When deciding on accepting this current offer, the KTSS algorithm allows us to assess the predicted graft survival probability of the current offer and the likelihood of receiving future kidney offers of different donor quality, HLA matching, and the time to the next offer. For example, the predicted graft survival of the current offer is 0.54 in 10 years. The next offer will likely occur in one month, be of more ideal quality, and have a higher predicted graft survival probability.

## Discussion

Considering the growing involvement of both clinicians and patients in collaborative decision-making, communicating complex information about the organ allocation process to patients, families and non-transplant health professionals remains a challenge. To meet this challenge, we implemented a KT match algorithm together with a visualization tool for predicting deceased donor kidney offers based on a well-defined allocation algorithm and 11 years of previous transplant data. This application provides predictive estimates for the anticipated wait time, donor quality, and the graft survival probability associated with subsequent kidney offers. In this study we have demonstrated how iterative simulation provides the basis to support individualized decision-making. Using a scenario-based approach, KTSS allows us to predict the estimated wait time required for the next kidney offer, the quality and the HLA matching if the current offer is declined. This information is crucial to making informed decisions (by the transplanting team, treating clinicians and patients) regarding the risk of complications associated with prolonged wait on the deceased donor waiting list while balancing against the risk of early graft loss in patients who have received suboptimal donor kidneys. By optimising offer acceptance, KTSS has the potential to reduce unnecessary declines and non-utilization of kidney offers, which could improve both survival and quality of life, and overall post-transplant outcomes. Additionally, KTSS can also improve organ allocation efficiency and equity by ensuring the donor kidneys are matched and transplanted to the most suitable transplant candidates.

Our KTSS system has several advantages. First, it provides clinicians and patients with easily visualised and interpreted information and predictions. This complements clinical decision-making and eases communication with potential recipients. Second, our platform is transparent and generalizable. Established based on the simKAP simulation framework, we are able to incorporate alternations of allocation algorithms that enables other researchers to establish new decision-making systems regionally. Third, our forecasting system has the capacity to provide sequential kidney offers rather than the next offer only.

KTSS has several limitations. The estimated wait time, donor quality, and HLA matching predictions are based on historical donor allocations and may not fully account for evolving organ donation and allocation policy trends. Additionally, HLA typing in the model primarily relies on single-field, low-resolution typing, which may not accurately reflect true molecular-level donor-recipient compatibility. The predictive models were trained using data from the ANZDATA registry, meaning their applicability to transplant candidates in other regions or healthcare settings may be limited. Furthermore, ongoing evaluation is essential to ensure that KTSS does not inadvertently prioritize certain subgroups or exacerbate existing inequities in transplantation access. Most importantly, formal validation and stakeholder engagement across the broader transplantation and donation sectors are necessary to refine the tool and optimize its integration into clinical practice.

As input data was sourced based on the previous allocation algorithm (prior to implementation of the new national deceased donor allocation algorithm in May 2020). We plan to validate the performance of KTSS system using the latest data derived from the newly implemented algorithm. The framework is flexible enough to adapt to any other allocation algorithm using different prior transplant data.

In summary, leveraging the combined power of simKAP and advanced statistical graphics, this innovative tool will significantly enhance decision-making for patients and clinicians. By offering intuitive visualizations that multiple potential kidneys offer with post-transplant prognostic insights, it empowers individuals to make informed choices about whether to continue dialysis in anticipation of a "high-quality" kidney or to accept the currently available offer.

## Supplementary Information

Below is the link to the electronic supplementary material.

Supplementary file1 (DOCX 20 KB)

## Data Availability

For the ANZDATA, data requests can be made through the ANZDATA registry, and access to the data source will require HREC approvals. Access to data can be contacted through the senior authors. The web interface is available at https://sydneybiox.github.io/KTSS_v2/.
