# Supplementary material for: A Kidney Transplant Support System for Patient-Clinician Shared Decision-Making
Source: J Med Syst. 2025 May 10;49(1):60. doi: 10.1007/s10916-025-02175-2 (PMC12064594; doi:10.1007/s10916-025-02175-2)
Supplement: Supplementary file 1 — Supplementary file1 (DOCX 20 KB) [file 10916_2025_2175_MOESM1_ESM.docx]

**Supplementary material**

*Data sources*

Data from the Australia and New Zealand Dialysis and Transplant (ANZDATA) registry was used to develop this decision-making tool. This data consists of all Australian candidates (n = 7740) on the waiting list who received a matched deceased donor kidney with starting replacement therapy time (KRT) between 30th June 2006 and 13th November 2017. Recipients with multiple kidney transplants were included, but multi-organ transplantations were excluded.

*Forecasting a potential sequence of donor kidney offers*

[A] Generate a collection of potential sequences.

To provide a sequence of potential kidney offers to a candidate on the waitlist, we applied our previously published kidney allocation simulation process (simKAP) [(Zhang *et al.*, 2023)](https://paperpile.com/c/PEfbac/Cps8U) to Australian deceased donor waiting list ~~[describe the data you use, check this is the correct terminology]~~ *(ANZDATA 40th annual report, accessed 29 Jan 2024).*and generated a sequence of simulated forecast of donor kidney offers for all candidates listed on the deceased donor kidney waiting list. simKAP was designed to generate recipient-donor pairs that mimic the real-life kidney allocation process. For every individual we generated a potential sequence of kidney offers by repeated application of simKAP, as shown in the *Figure 1*.~~and presented as a matrix.~~ *Results were saved as a matrix for visualisation purposes. To account for variabilities of results, we summarise our results as the average of repeated runs and also calculate the standard deviation as the variability provided in the table.* Here, each row corresponded to a single donor kidney offer for the potential candidate, and the columns consisted of 16 variables representing three separate categories.

- Recipient's characteristics, included recipient age at the time of offer, wait time, sex, blood group, presence of diabetes mellitus, PRA level, and the recipient's current state.
- Donor characteristics, including donor age, sex, kidney donor profile index (KDPI), blood group, donor’s state, presence of diabetes mellitus~~.~~
- Immunological measurements consisting of HLA A, B and DR mismatches.

[B] Individual estimated of potential sequence of donor kidney offers via matching algorithm.

For a given recipient, we develop a KTmatch algorithm based on the K-Nearest Neighbor (KNN) algorithm with KD-Tree from Ubilabs [(*kd-tree-JavaScript. JavaScript k-d Tree Implementation*, no date)](https://paperpile.com/c/PEfbac/3CYHZ) to identify one best matched candidate. The recipient and donor variables are used in the K-Dimensional Tree ( KD-Tree) algorithm were based on all 16 variables.

[C] Transplantation characteristics and statistical visualization

For a given identified donor kidneys offer, we calculated the candidate waiting time and the predicted probability of post-transplant graft survival associated with the potential offer.

(1) *Calculation of waiting time.*

Potential candidate waiting time is defined as the difference between *the* time of the potential kidney being offered and the time that the candidate started dialysis. This calculation is given by the following formula:

$$W_{ij}=D_{j}-R_{i},$$

where $W_{ij},$is the waiting time for the i^th^ recipient with the potential donor kidney offer j; and

$D_{j}$ is the date when the j^th^ donor' s kidney offer and $R_{i}$ is the waiting list start date of the i^th^ candidate.

(2) *Model for predicting post-transplant graft survival*.

We apply the Cox proportional hazard model given by the following formula to provide the predicted candidate’s post-transplant graft survival associated with a potential kidney offer

$H\left( t \right)=h_{0}\left( t \right)\times e^{X\beta} ,$

where $X$ is the vector contains10 covariates (recipient characteristics age, waiting time, ethnicity group, smoking status, recipient peripheral vascular disease, donor characteristics age, height, hypertension status, presence of diabetes mellitus and HLA-DR). These risk factors were chosen based on previous knowledge [(Calisa *et al.*, 2018)](https://paperpile.com/c/PEfbac/aQ1Z) . *Prediction for post-transplant graft survival for the 2nd, 3rd and 4th kidney offers are based on the cumulative time until the 2nd, 3rd and 4th offers, respectively.*

(3) *Graphical summary*

To assist with visualizing all the above information associated with organ acceptance decision-making, we developed a graphical visualization tool for the current and next offers that an individual candidate may receive. This infographics-like scatter plot visualizes the trade-off between the waiting time, characteristics of the kidney and the estimated post-transplant survival (EPTS), informed decision could be made by the relevant stakeholders. The size of each dots represents wait time, and the opacity of each dots represent the order of offers.

*Design of a web-based interactive decision-making platform*

The design of our interactive web-based platform to assist decision-making of offered kidneys aims to provide users the characteristics of the deceased donor kidneys being offered currently and the forecasted three sequential offers. *Our interactive platform was built on pre-generated results using methods demonstrated in section Forecasting a potential sequence of donor kidney offers*. Our application was built with the preferences of clinicians in mind, following a user-centric approach. By prioritizing their needs and workflows, we ensured that the application caters to their requirements effectively. Our design ensures that the application adapts seamlessly to different devices and screen sizes with emphasis on fast loading times and smooth interactions. To this end, we opted for a web server architecture over prototype R Shiny server to maximize accessibility for users. To safeguard patient privacy and data security, we utilize simulated data in the application. This approach protects the confidentiality of real patient information while still assisting decision-making for clinicians. The input and output of the webserver are:

*(1) Input* - The recipients and donors’ characteristics are required as the baseline input within the web-based platform. These characteristics are listed in Results section.

*(2) Output* - We presented the forecast for the next four offers and curated the comparison between the current and subsequent kidney offers as part of our output.

Our platform was developed using React.js, and it is hosted on ~~Netlify at~~ [~~https://ktss-exact.netlify.app/~~](https://ktss-exact.netlify.app/) *GitHub at:* [*https://sydneybiox.github.io/KTSS_v2/*](https://sydneybiox.github.io/KTSS_v2/)*.*
